# Past, present and future of Focused Ultrasound as an adjunct or complement to DIPG/DMG therapy: A consensus of the 2021 FUSF DIPG meeting

**DOI:** 10.1016/j.neo.2023.100876

**Published:** 2023-01-28

**Authors:** Kavya Parekh, Suzanne LeBlang, Javad Nazarian, Sabine Mueller, Stergios Zacharoulis, Kullervo Hynynen, Lauren Powlovich

**Affiliations:** 1 University of Virginia, Charlottesville, Virginia, USA; 2 Focused Ultrasound Foundation, Charlottesville, Virginia, USA; 3 Children's National Hospital, Washington DC, USA; 4 University of California San Francisco, San Francisco, California, USA; 5 Bristol-Meyers-Squibb, New York, New York, USA; 6 Columbia University Medical Center Department of Pediatric Oncology, New York, New York, USA; 7 Sunnybrook Research Institute, Toronto, Canada

**Keywords:** DIPG, Diffuse Intrinsic Pontine Glioma, DMG, Diffuse Midline Glioma, FUS, Focused Ultrasound, BBB, Blood-brain barrier, BBBO, Blood-brain barrier opening, SDT, Sonodynamic therapy

## Abstract

Diffuse Intrinsic Pontine Glioma (DIPG), now known as Diffuse Midline Glioma (DMG) is a devastating pediatric brain tumor with limited treatment options and a very poor prognosis. Despite more than 250 clinical trials aimed to treat children diagnosed with DMG, no curative therapies currently exist for this patient population. A major obstacle has been the intact blood brain barrier (BBB) which prevents most therapeutics from crossing into the tumor bed. Focused Ultrasound (FUS) is an emerging, noninvasive medical technology which has been shown in both preclinical and clinical research to disrupt the blood brain barrier safely and temporarily. FUS blood brain barrier opening has been studied in combination with chemotherapies in preclinical DMG models, and this technology is now being investigated in clinical trials for the treatment of pediatric brain tumors. Focused ultrasound has additional mechanisms of action, including sonodynamic therapy and radiation sensitization, that hold promise as future DMG therapies as well. This paper, largely based off the proceedings from a workshop held by the Focused Ultrasound Foundation in October of 2021, summarizes the current state of the field of focused ultrasound for DIPG/DMG, including preclinical, technical, and clinical summaries in addition to recommended next steps for continued advancement of the game changing technology of Focused Ultrasound.

## Introduction

Diffuse Midline Glioma (DMG), previously known as Diffuse Intrinsic Pontine Glioma (DIPG) is an extremely devastating pediatric brain tumor that is commonly diagnosed between the ages of 5-7 years old, with a greater than 90% mortality less than 2 years after diagnosis [1]. As the name implies, these malignant tumors infiltrate into and through the pontine portion of the brainstem. Given the sensitive neuroanatomical location of these tumors, surgical resection is not an option and radiation therapy is the only treatment that provides temporary clinical improvement [2].

There has been significant scientific discovery in the past decade which has led to the new diagnostic classification of DIPG to diffuse midline glioma, H3 K27M-mutant, based on the discovery of K27 mutations in a histone H3 gene [3]. However, despite this progress in classification and molecular subtyping, and innumerable clinical trials aimed to treat children diagnosed with DMG, no curative therapies exist for this patient population. A major obstacle has been the intact blood brain barrier (BBB) which prevents most therapeutics from crossing into the tumor bed. Evaluating the BBB permeability status is very difficult by current imaging modalities. The absence of contrast enhancement supports the hypothesis that the BBB in DIPG is intact at least for the most part. Recently, it has been shown in a human syngeneic BBB preclinical model that DIPG cells are not capable of disrupting the BBB integrity [4]. Additionally, a lower expression of tight junction proteins and reduced overall vascular density were noted in a study examining biopsy and post-mortem samples of DMG H3K27M, which could reflect a leaky BBB [5].

**Figure 1:**
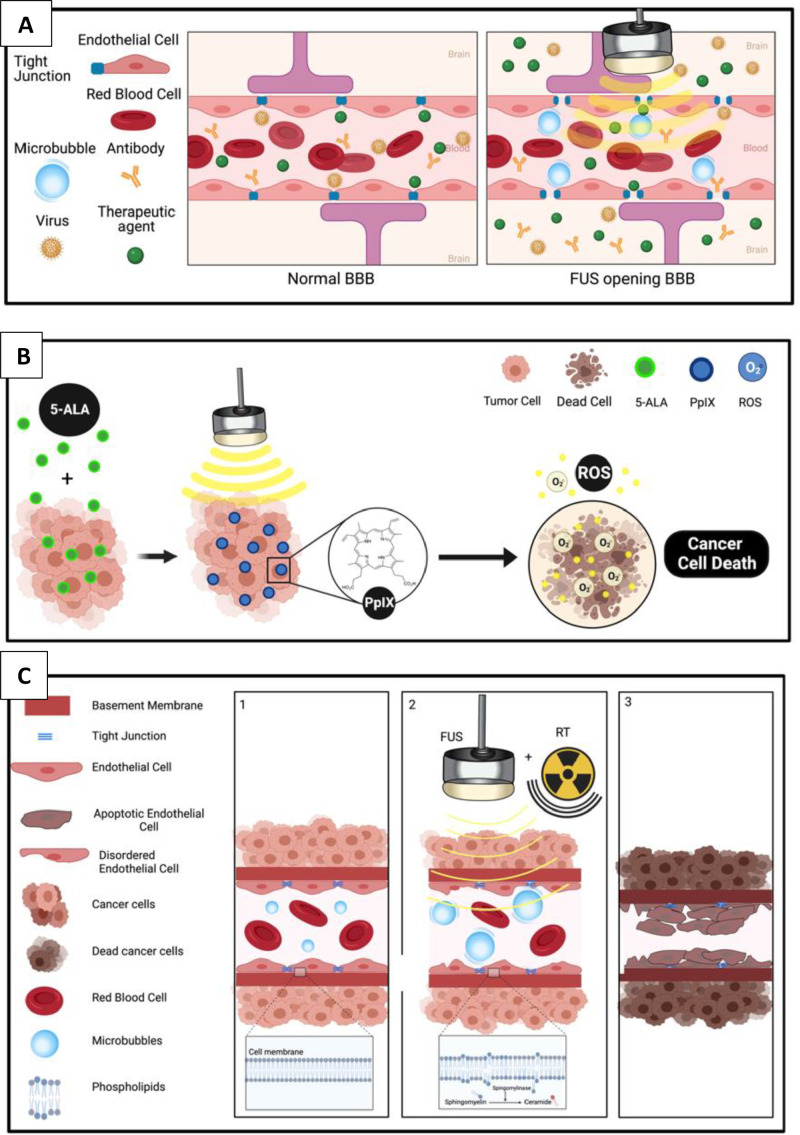
Focused Ultrasound Mechanisms of Action related to DIPG/DMG Therapy. (A) Blood Brain Barrier Opening: In the presence of the FUS beam, intravenously injected microbubbles oscillate inside the vessels and open the tight junctions, allowing therapeutics to diffuse into the targeted region. Not depicted here are the additional mechanisms of sonoporation and increased transcytosis which also occur with FUS mediated BBBO. (B) Sonodynamic Therapy: Intravenous injection of a sonosensitizer such as 5-ALA accumulates preferentially inside brain tumor cells such as DIPG. Conversion of the sonosensitizer into an active substrate (ie PpIX) induces tumor cell death. (C) Radiation Sensitization: The proposed mechanism of action involves ceramide-induced endothelial apoptosis, which subsequently enhances radiation by causing vascular disruption. Distortion of the endothelial cell membrane by oscillating microbubbles in the presence of the FUS beam releases ceramide which then causes platelet aggregation and thrombosis.

Focused Ultrasound (FUS) is an emerging, noninvasive medical technology which has been shown in both preclinical and clinical research to disrupt the blood brain barrier safely and temporarily. Preclinical research has reported the ability of FUS to open the BBB and enhance the delivery of chemotherapies into DMG models [6], [7], [8], [9]. Additionally, focused ultrasound has been shown to enhance the effects of radiation therapy and immunotherapy, also with BBB opening [10], [11], [12]. Lastly, sonodynamic therapy (SDT), where focused ultrasound is used to locally activate an inactive compound into one with cytotoxic effects on tumor cells, shows promise for patients with DMG. Figure 1 illustrates these mechanisms of action (Figure 1).

This article provides an overview of the current state of the field for Focused Ultrasound and DMG, including a review of preclinical research, dedicated focused ultrasound technology, and clinical trials and concludes with a roadmap suggesting where the field needs to progress to ensure that focused ultrasound treatments reach patients with DMG as safely and quickly as possible. Much of the content within this review is based on the proceedings from the Focused Ultrasound Foundation's *Focused Ultrasound for DIPG Workshop* [13], held in October of 2021, where some of the world's leading experts and clinicians in DMG convened to share their knowledge, experience and recommendations.

## Early Preclinical BBBO Findings

It is well established that focused ultrasound combined with intravenous injection of microbubbles leads to temporary, reversible disruption of the BBB. The local oscillation of microbubbles by FUS causes blood vessel distention as well as changes in endothelial cell cytoskeletons and cell-cell interactions. This leads to BBB disruption via three mechanisms: separation of the tight junctions, transcytosis, and sonoporation of the vascular membrane [14]. Many preclinical models have established the feasibility and safety of BBB-opening using FUS in conjunction with micro-bubbles [15], [16], [17]. After initial confirmatory studies, this capability was applied to the field of neuro-oncology, where the BBB is a known obstacle to the effective delivery of CNS-targeted chemotherapy. Many studies have found that therapeutic delivery via FUS-mediated BBB opening improved both delivery and survival rates in preclinical models of brain metastases and gliomas [15,[18], [19], [20], [21], [22], [23], [24]. Preliminary clinical trials for brain tumors have subsequently been conducted [25,26].

## Preclinical Studies in DMG Models

Additional studies have demonstrated that FUS-mediated opening of the pontine-specific BBB is possible and safe, even in the presence of a pontine tumor [27,28]. Importantly, no physiological, behavioral, or histological changes in the rodent models used were found. Moreover, using MR imaging as confirmation, it was demonstrated that the opening is reversible and spontaneously closes within 72 hours post-FUS [27]. BBB-opening with FUS was also shown to be safe in DMG models that were previously treated with whole brain radiation, confirming the clinical translatability of focused ultrasound in treating pontine gliomas [11]. However, radiation has also been shown to have a synergistic effect on the degree of BBB opening, indicating a need for further studies on sonification dose levels and schedules in cases of prior brain radiation [29].

### Doxorubicin

Subsequent studies concentrate on improving drug delivery to the central nervous system using FUS. Doxorubicin, a small chemotherapeutic agent that is cytotoxic to DMG patient cell lines, has been shown to have poor BBB permeability [11]. Studies have shown the utility of FUS BBBO to enhance the delivery of therapeutics to brain tumors [6], [7], [8]. For DMG/DIPG applications, it has been specifically demonstrated that FUS-mediated brainstem BBB opening in healthy mice allows for a 50-fold increase in Doxorubicin in the targeted area [30]. Additionally, FUS-mediated BBB opening in orthoptic DMG models revealed a four-fold increase in doxorubicin levels within the brainstem tumors, with a subsequent decrease in the volumetric tumor growth of tumors with low expression of Ki-67 [28]. However, there was no significant survival advantage found to be associated with an increased concentration of doxorubicin in the DMG models. A follow on study using a liposomal formulation of doxorubicin was conducted, hypothesizing that this would lead to increased doxorubicin exposure and thus improved survival, but again no survival benefit was found [31].

### Etoposide

Etoposide is another small chemotherapeutic drug that is often used to treat pediatric cancers but has notably limited efficacy against DMG. In syngeneic GBM models, etoposide delivery using FUS BBBO was not only increased, but showed survival benefits [9]. In murine models of pontine glioma, a similar five-fold increase in intra-tumoral etoposide levels with FUS BBBO was found [27]. Of note, the cell line used for this model was not an H3K27m tumor cell line. Physiological  functions were found to be stable after the procedure, and there was no increase in inflammation or tumor-specific hemorrhage. However, unlike the GBM models, the treated mice did not show any survival benefit.

Interestingly, FUS BBBO in DIPG models with both Doxorubicin and Etoposide have shown evidence of enhanced delivery of chemotherapeutic, but without improved survival. It is hypothesized that the lack of demonstrated survival benefit may be attributed to differences in the pharmacokinetics of these drugs in murine models compared to humans but requires further investigation [13].

### Antibodies

The intravenous manner of chemotherapy administration causes the additional issue of systemic toxicity, regardless of efficient local drug delivery to the brain. FUS in combination with microbubble-mediated intranasal delivery (FUSIN) has been proposed to minimize systemic chemotherapeutic exposure while maintaining the benefits of FUS-mediated drug delivery to the brain. Intranasal administration allows for direct nose-to-brain drug administration, and FUS-induced microbubble cavitation would continue to enhance the transport of intranasally administered agents to the FUS-targeted brain location. To test whether FUSIN safely and feasibly decreases systemic drug delivery, the biodistribution of IN administered gold nanoclusters (AuNCs) has been measured and compared to that of IV administered AuNCs. It was then found that IN administration showed significantly lower AuNC accumulation in the blood, lungs, liver, spleen, kidney, and heart compared to IV administration. Moreover, FUSIN reportedly enhances AuNC delivery compared to IN administration, again evidencing another technique for which FUS enhances local delivery [32]. Given these preliminary results, FUSIN was used to administer anti-programmed cell death-ligand 1 antibody (aPD-L1) to the brain in a glioma model. It was found that FUSIN enhanced the penetration depth and delivery efficiency of aPD-L1 and that aPD-L1 additionally colocalized with the tumor cells after FUSIN delivery. Notably for DMG applications, this study was conducted in a brainstem glioma model [33].

### Sonodynamic Therapy

The application of FUS for sonodynamic therapy for brain tumors originated from the well-known use of photodynamic therapy using light to activate a sonosensitizer for skin cancers [34].  As light cannot penetrate deep enough through the skull to effectively apply the technology for brain tumors, ample preclinical research has demonstrated that low intensity focused ultrasound can activate systemically administered sonosensitizers such as 5-ALA, that preferentially accumulate in tumors and is converted into PpIX via the heme pathway and produces cytotoxic agents to immediately induce cell death inside the gliomas and clinical studies are now enrolling patients.  The cytotoxic reaction only occurs when FUS is combined with a sonosensitizer and neither FUS nor the sonosensitizer alone is capable of inducing cell death individually [35]. The safety of this technique has been validated in a large animal preclinical study [36] and clinical trials of SDT combined with 5-ALA are now underway (NCT05370578, NCT04845919, NCT05123534).

A prerequisite for sonodynamic therapy to be effective is, of course, evidence that the sonosensitizer accumulates in tumor cells. It has been widely demonstrated that 5-ALA has preferential uptake in gliomas [37], pediatric brain tumors [38,39], and DIPG [40]. At the FUSF DIPG workshop, Dr. Javad Nazarian's team shared that not only does 5-ALA, an FDA approved sonosensitizer, accumulate in higher amounts in DMG cells compared to C6 rat glioma models, but also that the DMG cells retain the 5-ALA and produce PpIX for longer amounts of time [13].  5-ALA is administered orally or through IV injections. A higher oral dose is required to achieve the same tissue concentration compared to the IV route and can lead to systemic complications. Technical research has reported that longer burst lengths during the FUS exposure leads to a greater therapeutic effect although this results in smaller treatment volumes.  The exact sonication parameters to optimize SDT for DMG remains under investigation.

The proposed mechanisms by which FUS induces the cytotoxic event is summarized in a recent review article by Bunevicius, et al. [41].  After a sonosensitizer such as a porphyrin derivative or xanthene dye accumulates in the tumor, the focused ultrasound beam can activate the sonosensitizer to produce free oxygen radicals that induce cell death through several potential mechanisms of action. First, the generation of light by collapsing microbubbles during FUS induced cavitation can activate the sonosensitizer and produce free radicals.  Secondly, pyrolysis may occur in the proximity of the collapsing microbubbles creating reactive oxygen species.  In addition, the sonosensitizer combined with the mechanical effects of the FUS beam can cause physical destabilization of the plasma membrane leading to cell death. However, the pressure amplitude levels used in these studies are lower than those reported for induction of cavitation in in-vivo tissues and thus the mechanism of activation is still not completely documented.

### Radiation Enhancement

As previously reported, FUS BBBO is safe in animals that have undergone radiation [11]. Using FUS to complement or enhance radiation is also being investigated. Initial studies showed ultrasound exposure with intravascular diagnostic microbubble contrast agent increases radiation's efficacy in both prostate and bladder cancer xenografts in mice models [12,42]. The proposed mechanism of action involves ceramide-induced endothelial apoptosis, which subsequently enhances radiation by causing vascular disruption. Further studies on ultrasound-mediated radiation enhancement specific to DMG and other gliomas would thus be of interest. Given the vital nature of the pons and other brain structures, it will be important to determine how the vascular collapse created by the radio sensitization may affect healthy, normal tissues.

Evidenced by the preclinical body of work described above and summarized in Table 1, focused ultrasound holds promise as an adjunct or complement to DIPG/DMG treatment through blood brain barrier opening, radiation enhancement and sonodynamic therapy.

**Table 1:** Focused ultrasound preclinical laboratory studies relevant to DMG.

Reference	Treatment	Animal Model	Repot synopsis
Treat et al. 2007 [8]	Doxorubicin	Wild-type rats	MRI-guided focused ultrasound with preformed microbubbles alongside Doxorubicin gave minimal tissue damage at 886 +/- 327 ng/g tissue while 5,366 +/- 659 ng/g had more significant more significant tissue damage. Drug accumulation with FUS remained lower across all paired samples.
Alli et al. 2018 [30]	Doxorubicin	Sprague Dawley rats NSG mice, no tumor	MRgFUS at rat brainstem gave no significant histological or functional deficits. 50-fold increase in Doxorubicin concentration in the mouse healthy brain stem.
Ishida et al. 2021 [28]	Doxorubicin	DMG murine orthotopic mouse model	4-fold higher increase in drug delivery to tumors. Volumetric tumor growth decreased in tumors that also had decreased Ki-67 expression.
Treat et al. 2012 [43]	Liposomal Doxorubicin (DOX)	Sprague-Dawley rats with glioma in left frontal lobe	Reduced tumor growth. Increased median survival.
Wei et al. 2021 [9]	Etoposide	GBM syngeneic mice model	8-fold increase in etoposide concentration in brain tumor tissue. 3.5 fold increase in brain tumor-to-serum ratio of etoposide. Decreased tumor growth by 45% and increased survival by six days.
Englander et al. 2021 [27]	Etoposide	DMG syngeneic orthotopic mouse model	5-fold increase in drug delivery with FUS. Physiological functions remained stable during FUS-mediated BBO. No difference in weight loss, tumor-specific hemorrhage, or inflammation. No survival benefit found.
Tran et al. 2012 [42]	Radiation	Human HT-1376 Bladder cancer xenografts in severe combined immuno-deficient mice	Ultrasound-microbubble treatments combined with radiation gave enhanced cell death, vascular normalization and areas of fibrosis. Both low and high microbubble concentrations with radiation gave increased tumor death.
Czarnota et al. 2012 [12]	Radiation	Human PC3 prostate cancer xenograft mouse model	Ultrasound-microbubble vascular perturbation combined with radiation give 10-fold greater cell kill.
Ye et al. 2018 [32]	Intranasal delivery of gold nanoparticles (AuNCs)	C57BL/6 female mice	IN administration induced significantly lower AuNC accumulation in the blood, lungs, liver, spleen, kidney, and heart compared with IV injection. Higher concentration of AuNC were found in the stomach, suggesting that the nanoclusters to be excreted from the animal through feces, thus minimizing toxicity. FUS+IN delivery enhanced delivery to the FIS-targeted brainstem area. No gross behavior changes noted.
Ye et al. 2021 [33]	Intranasal delivery of anti-programmed cell death-ligand 1 antibody (aPD-L1)	Orthoptic implantation of murine glioma cells (GL261) at the brainstem in mice model	FUSIN enhanced the penetration depth and delivery efficiency of aPD-L1. aPD-L1 colocalized with the tumor cells after FUSIN delivery.
Wu SK et al. 2019 [35]	5-ALA (Sonodynamic therapy)	Sprague-Dawley rats; intracranial C6 glioma cells	Inhibition of tumor growth (evaluated using weekly MRI) and survival vs. FUS alone or 5-ALA alone FUS and 5-ALA alone did not improve animal survival Lesser Ki67 expression and TUNEL overexpression

## Focused Ultrasound Technology

There are four therapeutic ultrasound companies in the clinical treatment stage that focus on neurologic indications, with blood brain barrier opening and other low intensity mechanisms heavily prioritized (Table 2). Three of the four companies are using their device to treat pediatric brain tumors in clinical trials.

**Table 2:** Clinical Therapeutic and Focused Ultrasound Devices being used in brain tumor treatments.

Company	# of Ultrasound Elements	Real time image guidance technique	Procedure location	System
Delsona	1	Neuronavigation with prior MRI	Office (examination room)	
Insightec	1024	MRI	MRI Suite	
Carthera	9	None	Operating Room (implantation) Office (treatments)	
NaviFUS	256	Neuronavigation	Office (examination room)	

### Delsona

The first in human clinical trial to open the BBB in the infratentorial brain region and potentially increase drug delivery in patients with DMG utilizes the Delsona device. Neuronavigation guides the single element low intensity FUS transducer into the targeted regions along the inferolateral margins of the DMG tumors.  Optical neuronavigation enables the procedure to be performed in an examination room using a previously performed MRI, optical guidance, and virtual fiducial markers to guide the 10-cm wide transducer into position, allowing BBBO in the preplanned location. The patient's head remains stable in a chin strap, like one used during an ophthalmologic exam, and thus no headframes or invasive maneuvers are needed. The FUS sonications are delivered during intravenous microbubble injections to achieve stable cavitation and BBBO. Real-time US monitoring for cavitation is performed that includes 2D cavitation mapping showing where the FUS spot is located at any time during the sonication while also indicating the bubble perfusion and activation [44,45]. There is currently one clinical trial utilizing this device for blood brain barrier opening in DMG patients, which is on hold, but will re-open soon. The benefit of this device for the pediatric population is that it does not require head stabilization with pins, and thus is less traumatic to the patient and does not require sedation.

### Insightec

Insightec Ltd. is a focused ultrasound device company that specializes in both high intensity and low intensity focused ultrasound systems for the brain. These systems use MRI guidance for pre-treatment planning and intraprocedural monitoring. For the low intensity system, which is the one employed for BBBO, the patient is placed inside an MRI machine with the head secured noninvasively with either subcutaneous plastic screws or a bite plate.  A helmet housing a 1024 element low intensity FUS transducer is placed around the patient's head.  Real time MR images along with the software allows for precise targeting of the region of interest in and around the tumor, activating a percentage of the ultrasound elements to effectively allow for focusing of the beam through the skull.  Real time MR images ensure BBBO is limited to the targeted region. In addition, cavitation threshold detection and spatial mapping monitor for stable cavitation (safety) and homogeneous BBBO (efficacy in targeted drug delivery) [46]. Post contrast MR images can be performed after the procedure to ensure BBBO in the target area. This device is currently being used in multiple adult clinical trials of BBBO for Glioblastoma [47]. This same machine, with slightly different technical parameters, is being used for sonodynamic therapy in patients with DMG (NCT05123534) as the FUS beam will activate a sonosensitizer, 5-ALA, which selectively accumulates in brain tumors, to produce free oxygen radicals causing cell death in vivo. The benefit of this system is that it can perform large volume opening compared to the other devices. The drawback is that it required head stabilization and thus patient sedation.

### Carthera

Although not currently used to treat DIPG/DMG patients, another ultrasound device that has been used in clinical trials for BBBO in adult brain tumor patients is now recruiting pediatric patients with supratentorial gliomas.  The SonoCloud-9® device (Carthera, Lyon, France), is comprised of 9 ultrasound transducers attached to a titanium mesh that measures 5.5 × 5.5 cm and can replace a bone flap.  The transducers can be implanted using neuronavigation during surgical removal of a tumor, if applicable.  Activation of the system can be performed without image guidance, at the time of infusion of chemotherapy (in the infusion suite).  To activate the SonoCloud device, a transdermal needle is connected to an external power source that is controlled via a touchscreen interface and the device is activated for a duration of 4.5 minutes.  This easy-to-use system allows for repeated treatments in an office-based setting while minimizing treatment time as no imaging or targeting time is required. There is one clinical trial (NCT05293197) investigating the safety and feasibility of this device for the treatment of supratentorial brain tumors in the pediatric population. Additionally, an earlier version of the SonoCloud device has been tested in the adult population through clinical trials, and has been found to be safe and well tolerated [25].

### NaviFUS

Another device that is currently being tested for use as a BBB opening device in patients is the NaviFUS System (Taipei City, Taiwan). The focused ultrasound array is Neuronavigator-guided and has 256 transducer elements with active cavitation monitoring and control [48]. The first in human trial conducted using the NaviFUS System (NCT 03626896) was conducted in Taiwan in recurrent GBM patients. This study proved safety and feasibility of BBBO in recurrent GBM patients using the NaviFUS device (publication of results is pending). Although there are currently no trials treating pediatric patients with this device, it could be used for DMG treatments with BBB opening and sonodynamic therapy. Much like the Delsona device, this system does not require head stabilization and thus no sedation is required.

## Clinical Trials Involving Focused Ultrasound for DMG

Given the compilation of promising preclinical evidence in addition to the growing safety and efficacy data using FUS for BBBO in Glioblastoma patients, Focused Ultrasound clinical trials for DMG patients are now enrolling, with additional trials opening soon (Table 3).

**Table 3:** Focused Ultrasound for DIPG/DMG clinical trials.

Title	NCT	Status	Location	Size	Device	Mechanism of Action	Drug
Non-Invasive Focused Ultrasound (FUS) in Children With Progressive Diffuse Midline Glioma (DMG)	NCT04804709	On hold	New York, USA	15	Delsona	BBBO	Panobinostat
A Safety and Feasibility Study to Evaluate Blood-Brain Barrier Disruption Using Exablate MR-Guided Focused Ultrasound in Combination with Doxorubicin in Treating Pediatric Patients with Diffuse Intrinsic Pontine Gliomas	NCT05630209 NCT05615623	Not yet recruiting	Washington DC, USA Toronto, Canada	20	InSightec	BBBO	Doxorubicin
A Phase I/II Study of Sonodynamic Therapy using SONALA-001 and ExAblate 4000 Type 2 in Patients with DMG	NCT05123534	Recruiting	Phoenix, USA California, USA Washington DC, USA	18	InSightec	SDT	IV 5-ALA

There are three clinical trials utilizing FUS BBBO and one clinical trial employing sonodynamic therapy that are in process or beginning recruitment soon. The first in the world clinical pilot trial of focused ultrasound for DIPG/DMG began enrolling patients at Columbia University in 2020 using the Delsona device combined with oral Panobinostat. Researchers at Columbia have shared that the first three patients were successfully treated in this clinical trial with no serious adverse events related to the focused ultrasound treatments [13]. However, this clinical trial has been placed on hold after Panobinostat was removed from the market for clinical use. The team plans to re-open the trial with Etoposide soon. The blood brain barrier opening clinical trials at Children's National Hospital and Sunnybrook Health Sciences Centre have recently begun enrollment. Both institutions are using Doxorubicin and the trial designs are complementary to one another.

The Sonodynamic Therapy clinical trial at Children's National (NCT05123534) is examining the safety and efficacy of SDT with IV 5-ALA (SonALA-001) in DIPG patients and is actively recruiting.

## Future Opportunities

There are specific areas within the field of Focused Ultrasound where further scientific inquiry will continue to advance the technology forward for DMG patients, in the preclinical, technologic, and clinical spaces.

### Preclinical

An outstanding question that exists within the preclinical space is: although tumor size was decreased with FUS BBBO + etoposide [27] and doxorubicin [28], why was there no survival benefit in either of these studies? As discussed at the FUSF DMG workshop, it is hypothesized that this is due to the pharmacokinetics of certain chemotherapeutics in the murine population. To further evaluate this hypothesis, additional studies should evaluate the murine pharmacokinetics of various chemotherapeutic agents to improve the translation of preclinical studies into effective clinical trials [13].

Several DMG in vivo models exist that may be suitable for FUS studies.  These include PDX models established by orthotopic injection or human primary cells, or syngeneic models developed by genetic engineering to introduce DMG driver genes at pre or postnatal developmental stage in mice [49], [50], [51], [52].  While all the existing models recapitulate DMG biology, few characteristics including tumor spread, engraftment, and survival time varies across these models.  Thus, selecting an in vivo DMG model for FUS studies should highly depend on question to be asked.  For example, while all existing models are suitable for assessing BBB opening, and drug distribution; assessment of immune modulation post FUS may be limited to immune competent mice only. On the other hand, if drug efficacy in increasing survival is being studies, an investigator may choose more than one model to represent DMG heterogeneity and tumor cell distribution across brain parenchyma.

A large, immunocompetent animal model will also improve preclinical evidence. Supported by the SebastianStrong Foundation, a team of researchers at Children's National Hospital, Columbia University and Virginia Tech have formed the first interdisciplinary FUS translational team with a focus on DMG.  The team has since formed a specialized working group (Focused DMG) that encompasses institutions across Europe and the US. The goal of the Focused-DMG team is to establish standards for preclinical and clinical FUS applications and translation of their findings through PNOC DMG clinical trials.

### Technologic

All the current ultrasound devices have been developed and tested for the treatment of adult patients and are the first-generation devices to test the feasibility, safety, and effectiveness of the BBBO in clinical settings. Thus, they are far from optimal, and many technical improvements have been developed and tested in laboratory and preclinical experiments. The treatment of a diffuse tumor in the pons of children with BBBO requires further considerations. First, due to the critical nature of the normal tissue in the pons, the ultrasound exposure control must be precise and well controlled. Even a small volume over exposure can have serious consequences. Second, related to the first, the target site is close to bony structures that can reflect ultrasound and cause standing waves that can cause uneven and unexpected exposure levels in the tissue. Finally, the skull properties of children are largely unknown and can lead to uncertainties in the treatment planning and execution.

Opportunities for technologic and/or feature improvements to existing clinical FUS systems for DMG treatments include the following:

• Three-dimensional spatial monitoring and control of the bubble activity
• Treatment planning that considers standing waves and allows exposure optimization
• A method of quantification of skull properties prior to the treatment
• Incorporation of a technology/methodology to better quantify drug delivery during or post-FUS BBBO
• A frameless system that does not require full head immobilization
• A portable system outside of the MRI environment

### Clinical

At the time of this journal submission, focused ultrasound blood brain barrier opening combined with chemotherapy has been administered in very few DIPG patients. Over the next 1-2 years, the safety and feasibility of this therapy should be established through ongoing and forthcoming clinical trials. With each clinical trial, a different therapeutic may be administered along with FUS-BBBO and so it is important to note that the systemic pharmacokinetics of drugs (like etoposide and doxorubicin), as well as tissue accumulation should be predicted using PKPD modelling, to design optimal schedules on dosing, infusion scheme and timing around FUS-BBBO.

In addition to the ongoing and forthcoming FUS-BBBO combined with chemotherapies clinical trials for DIPG, there are other modalities involving FUS that may have an impact on DIPG in the future: liquid biopsy and immunomodulation/immunotherapy.

### Liquid biopsy

Liquid biopsy is a burgeoning methodology for detecting and monitoring cancers outside the central nervous system, yet brain tumors such as DMG are typically difficult to detect as the BBB prevents biomarkers from entering the bloodstream. As previously discussed, the BBB remains mostly intact with DMG, as there is typically no enhancement on MRI and thus to diagnose and follow DMG, MRI is often utilized.

Notably, tumor DNA from DMG cells has been detected in the cerebrospinal fluid (CSF) and treatment response can be measured by monitoring the allele fraction of mutations in the cfDNA [53,54]. The allele fraction change over time has correlated with progression free survival in the CSF and to a lesser degree in plasma [13]. Additionally, it has been shown that increases in cfDNA predict tumor progression 30-60 days in advance and patterns in allele fraction change can help differentiate true progression from pseudo-progression. However, repeat CSF analysis via lumbar puncture also carries risk and thus having the ability to sample the blood could dramatically improve the safety monitor disease over time.

Focused ultrasound could be used to temporally amplify the cfDNA in plasma and CSF by opening the BBB in patients with DMG to allow for improved detection of tumor biomarkers and cellular mutations to guide precision medicine. The risk of causing metastatic disease is unlikely with DMG as these cells require the brainstem environment to grow.  DMG clinical trials should incorporate standardized protocols for CSF and plasma collection as well as the accumulation of harmonized data on MRI scans, clinical outcomes, and specific analytes.

### Immunotherapy

Promising immunotherapy drugs have been approved to treat a variety of different cancers, with the potential to achieve remission and even cure certain patients. Focused ultrasound has emerged as a modulator of the tumor microenvironment, which can result in the enhanced effectiveness of immunotherapeutics and turn “cold” tumors into “hot” tumors [55].

The tumor microenvironment of DIPG/DMG is characterized by fewer immune cells and less inflammatory myeloid cells that many tumors, even when compared with adult glioblastoma (GBM) [56]. Better understanding of T cell homing across the BBB should help to formulate improved strategies to enhance an immune response to these difficult to treat tumors and might elucidate how focused ultrasound can help to convert these tumors into ones that are responsive to immune therapies.

## Conclusion

DIPG/DMG is a devastating pediatric high-grade central nervous system tumor with no effective treatment options and poor prognosis. Focused ultrasound has been shown to safely and non-invasively open the blood brain barrier in both preclinical and clinical settings to enhance the delivery of therapeutics to brain tissue, including brain tumors such as GBM and DMG. Clinicians and researchers are currently investigating FUS BBBO in clinical trials and further preclinical work should continue to elucidate potential combination medications and standardized methodologies. Sonodynamic therapy is another treatment modality involving focused ultrasound where preclinical evidence is promising for the potential treatment of DMGs, and a pilot clinical trial is underway. There is consensus that some of the features of focused ultrasound devices could be improved to enhance the patient experience and treatment efficacy and these suggestions should be seriously considered. Radiation enhancement, liquid biopsy, and immune modulation are three additional focused ultrasound mechanisms that deserve attention and further investigation in the DMG patient population. There is an abundance of work to be accomplished with Focused Ultrasound for DMG, but the payoff of improving the lives of children stricken with devastating brain tumors will be well worth the investment.

## CRediT authorship contribution statement

**Kavya Parekh:** Writing – original draft, Writing – review & editing. **Suzanne LeBlang:** Writing – original draft. **Javad Nazarian:** Writing – review & editing. **Sabine Mueller:** Writing – review & editing. **Stergios Zacharoulis:** Writing – review & editing. **Kullervo Hynynen:** Writing – review & editing. **Lauren Powlovich:** Conceptualization, Writing – original draft.

## Declaration of interests

The authors declare that they have no known competing financial interests or personal relationships that could have appeared to influence the work reported in this paper.
